# The relationship between individual differences and Spanish achievement among Chinese undergraduate students

**DOI:** 10.3389/fpsyg.2025.1622521

**Published:** 2025-08-07

**Authors:** Qi Lu, Eulalio Fernández Sánchez

**Affiliations:** ^1^School of International Studies, Wenzhou Business College, Wenzhou, China; ^2^Department of English and German Philologies, University of Cordoba, Cordoba, Spain

**Keywords:** Expectancy-value motivation, language anxiety, social identity, Spanish achievement, learning Spanish as a foreign language (ELE)

## Abstract

This study investigates how three individual differences (language anxiety, Expectancy–Value Motivation, and social identity) predict academic performance in Spanish as a foreign language among a sample of 317 Chinese undergraduate students majoring in Hispanic Philology. A designed questionnaire was used to collect all the data from the participants. The results demonstrated that while Chinese learners of Spanish generally possess a high level of value motivation, especially utility value, they showcase a medium level of expectancy. Additionally, participants show a medium-low level of language anxiety in the institutional context of Spanish as a foreign language (ELE) and experience a positive trend towards changes in their social identity, particularly when it comes to self-confidence. Moreover, achievement value and self-confidence emerge as positive predictors of Spanish achievement, while language anxiety is the only negative predictor. Based on the above, limitations and future lines of research are also discussed.

## 1 Introduction

Individual differences in second language acquisition (SLA) encompass student traits and characteristics that influence the processes, behaviors, and outcomes of acquiring a second language (L2) (Ortega, 2009; Fernández Sánchez, 2018; Li et al., 2022). Among these differences, language anxiety and motivation have garnered significant attention in SLA, with studies consistently identifying language anxiety as a negative predictor of the foreign language teaching-learning process (Gardner, 2007; Oxford, 2015; Busse, 2017). However, motivation is often identified as a positive factor (Doğan and Tuncer, 2016; Horwitz, 2017). From a sociocultural perspective, social identity has been considered an important factor in this field since the process of learning an L2 entails, familiarizing oneself with a completely different culture, population, and society (Norton, 2013; Schumann, 1986).

A comprehensive literature review reveals certain limitations in studies addressing these three factors. Specifically, it is found that motivation research predominantly relies on Gardner’s (1985) socio-educational model or Dörnyei’s (2008) L2 Motivational Self System; both are critiqued for methodological rigidity. Additionally, language anxiety is often measured using the Foreign Language Classroom Anxiety Scale (FLCAS) developed by Horwitz et al. (1986). However, since the scale was originally designed for English learners, its application to other languages raises concerns about its construct validity and cross-linguistic appropriateness. On the other hand, social identity theory, as proposed by Norton Peirce (1995) and later expanded by Norton (2013), is primarily examined in immigrant contexts. However, learners’ social identity in institutional contexts should also be taken into account, as it contributes to how they position themselves throughout their language learning process and may influence their motivation, language anxiety, and overall learning outcomes. Taken together, these observations suggest both the theoretical frameworks and methodological approaches in existing research may be somewhat outdated.

To address these limitations, the present study introduces three key updates. First, it adopts the Expectancy–Value Motivation (EVT) proposed by Eccles and Wigfield (2020), which offers a more current and flexible framework than earlier models. The EVT plays a crucial role within the framework of control-value theory (CVT) in explaining the emotional roots of academic performance. By focusing on learners’ sense of control and the value they attach to learning tasks, it helps to explain how motivation shapes academic emotions, which can influence engagement and achievement. Second, in place of FLCAS, this study employs the Spanish as a Foreign Language Anxiety Scale developed by Moneypenny and Aldrich (2022). This scale provides a more precise and context-sensitive assessment of language anxiety among learners of Spanish. Third, this study draws on Luo’s (2019) institutional social identity model. This approach better accounts for the identity shifts that occur in academic contexts and serves as a better fit for understanding how Chinese university students construct and negotiate their identities while studying Spanish as a foreign language.

Although recent studies have increasingly focused on these three affective variables in the context of Spanish as a Foreign Language (ELE), their number remains limited (Minera, 2010; Luo, 2019; Clouet, 2020; Wada, 2021). Moreover, few investigations have explored potential relationships between internal or external factors and academic performance in Spanish (Rabadán Zurita and Orgambídez Ramos, 2018a,b). Therefore, the present study aims to fill this gap by examining Chinese university students learning ELE.

This article is structured as follows: Section 2 reviews relevant theories on motivation, language anxiety, and social identity. Section 3 outlines the methodology, including research questions, participant details, instruments, and procedures for data collection and analysis. Sections 4 and 5 present results and discuss their implications. The final section presents the conclusions, along with the study’s limitations and suggestions for future research directions.

## 2 Literature review

### 2.1 Expectancy-Value Motivation

Motivation has played a fundamental role in second language acquisition (SLA) and has remained a central topic since the 1970s. Among the various theories, Gardner’s (1985, 2007) socio-educational model of motivation and Dörnyei’s (2009) L2 Motivational Self System have received significant attention. However, both have faced criticism in recent years. Specifically, the former has been criticized for its overreliance on integrative and instrumental motivation, with limited grounding in educational psychology or empirical evidence (Oga-Baldwin et al., 2019). The latter model combines three key components: cognition, affect, and motivation. It integrates cognition, affect, and motivation, linking dynamic motivation with L2 learners’ identity. However, the model suffers from serious methodological flaws, primarily due to a lack of basic statistical rigor (Al-Hoorie, 2018). This limitation undermines the model’s ability to reliably predict language proficiency (Nagle, 2021).

To address these shortcomings, the EVT (Eccles and Wigfield, 2020) has recently been widely applied in the field of SLA as a compelling alternative. Originally proposed by Atkinson (1957) and developed by Wigfield and Eccles (2000) and Eccles and Wigfield (2020), this model suggests that an individual’s motivation to perform an academic task rests on two psychological factors: the expectation of success and the values associated with the task (Dong et al., 2022). Expectations of success refer to an individual’s perceived competence in performing various learning tasks in specific domains, extending across past, present, and future contexts. Meanwhile, task values represent the subjective reasons that drive a learner to choose and persist in a particular learning task or activity, and these values consist of four subtypes: achievement value, intrinsic value, utility value, and cost value. Achievement value relates to the importance of performing well on a task or activity and is tied to personal goals; meanwhile, intrinsic value is reflected in the internal gratifications (such as enjoyment and rewards) that the individual gains from engaging in and completing a task or activity. As for utility value, it refers to the individual’s assessment of the usefulness after completing the task or activity, considering current or future goals. Lastly, cost value refers to the individual’s evaluation of the amount of effort, opportunities, and emotional burden required to complete a task or activity.

Several empirical studies (e.g., Loh, 2019; Wan, 2019; Nagle, 2021; Dong et al., 2022) have revealed that this model can be used to explain and predict L2 students’ choices, effort, persistence, and academic performance, establishing a positive relationship between expectations and language proficiency. It should be noted, however, that research on EVT remains limited and has largely focused on the context of English as a foreign language. Thus, this observation needs to be verified with further empirical evidence in the context of other languages (Eccles and Wigfield, 2020).

### 2.2 Language anxiety

In addition to motivational constructs, recent research has emphasized affective variables in SLA, particularly language anxiety. Language anxiety refers to “the feeling of tension and apprehension specifically associated with second language contexts, including speaking, listening, and learning” (MacIntyre and Gardner, 1994, p. 284). Rubio (2004) noted that this emotional state can arise from physiological, cognitive, and assertive sources. Given that this specific anxiety primarily occurs in institutional settings, Horwitz et al. (1986) defined language classroom anxiety as “a distinct complex of self-perceptions, beliefs, feelings, and behaviors related to classroom learning arising from the uniqueness of the language learning process” (p. 128). This definition has become the most widely used and well-known theory of language anxiety in SLA research.

These authors classify language anxiety into three specific categories: communication apprehension, test anxiety, and fear of negative evaluation. Subsequent studies have identified additional dimensions that reflect the evolving complexity of language learning environments. Young (1991) emphasized the role of teacher–student interaction, while Gregersen et al. (2014) highlighted classroom competition as an important source of anxiety. Other research has pointed to communicative confidence (Park, 2014) and fear of failure (Toyama and Yamazaki, 2019) as further contributing factors. Moreover, FLCAS (Horwitz et al., 1986) has become the most reliable and widely used instrument for measuring foreign language learners’ anxiety in classroom settings (Teimouri et al., 2018). Building on this scale, Moneypenny and Aldrich (2022) developed a Spanish-as-a-foreign-language anxiety scale by adapting the Arabic Foreign Language Anxiety Questionnaire (Dewaele and Al-Saraj, 2015) for effective use in the ELE context.

Empirical research has consistently shown that language anxiety exerts a negative influence on students’ academic performance in the foreign language classroom (Horwitz, 2017; MacIntyre, 2017). It is also inversely correlated with linguistic proficiency (Aida, 1994; Woodrow, 2006; Shao et al., 2019; Zhang, 2019), as well as other individual differences, such as motivation and enjoyment (Dewaele and Li, 2022; Jiang and Dewaele, 2020; Lou and Noels, 2020).

### 2.3 Social identity

Complementing cognitive and affective approaches, the social dimension of language learning has gained attention through research on social identity. In SLA, social identity refers to how learners understand their relationship to the world through language. From a sociocultural perspective, social identity has arguably been the most extensively studied subject in SLA (Darvin and Norton, 2015; Almarza et al., 2012). It shapes how learners perceive themselves and their place in society as they acquire a new language. Lambert (1967) suggests that developing linguistic competence in both the first and second languages is associated with changes in the social identity of L2 learners. Building on this foundation, Schumann (1986) introduced the acculturation model, which emphasized social distance as the crucial factor. He argued that reducing this social distance, through greater contact with and identification with the L2 culture, facilitates language acquisition.

Meanwhile, Norton (2013) further advanced the conceptualization of social identity by framing it as the dynamic relationship among the individual, language, and society. She defined it as “how a person understands his or her relationship to the world, how that relationship is structured across time and space, and how the person understands possibilities for the future” (p. 45). In SLA, this means that L2 learners must invest in their second language to understand their social position within the L2 community—a position that may or may not be deemed legitimate by L2 speakers—to subsequently speak, read, and write in that language (Norton Peirce, 1995).

Although early studies focused on immigrant contexts, social identity has been adopted in language education settings (Norton and Gao, 2008). In this context, Luo (2019) introduced the concept of identity change, based on a bilingual orientation (e.g., Gao et al., 2005). This model posits that identity change in L2 learners is a byproduct of the L2 learning process and is reflected in the development of their L1 proficiency. Luo identified four types of identity change: self-confidence (where L2 proficiency enhances learner confidence); subtractive change (where L2 learning diminishes L1 identity); additive change (where L2 strengthens L1 identity); and productive change (where both L1 and L2 identities are mutually enriched). These changes are often reciprocal and may extend to other personal and academic dimensions. This framework is particularly relevant for the current study, as it avoids the immigrant bias of prior theories and captures academic-specific identity shifts (e.g., exam performance pressures). Furthermore, it aligns with China’s utilitarian approach to foreign language learning (Dong et al., 2022).

## 3 Methodology

### 3.1 Research questions

In recent years, the number of Spanish Philology departments in Chinese universities and the total number of students studying Spanish as a foreign language have reached 105 and 34,823, respectively (Instituto Cervantes, 2024). Compared to the year 2000, these figures reflect a nearly sixfold increase in the number of institutions and more than a twentyfold rise in student enrollment over a 25-year period (Lu, 2023). Despite this significant expansion, there remains a lack of empirical research addressing individual differences among Chinese university students of Spanish. Consequently, it is crucial to analyze the language anxiety, motivation, and social identity of Chinese students of Spanish to explore the potential effects of these factors on their academic performance in Spanish. Therefore, the present study aims to answer the following research questions:

*RQ1*: What is the profile of individual differences (language anxiety, Expectancy–Value Motivation, and social identity) among Chinese undergraduates majoring in Hispanic Philology?

*RQ2*: To what extent do language anxiety, Expectancy–Value Motivation, and social identity predict the academic performance in Spanish of Chinese undergraduate students majoring in Hispanic Philology at Chinese universities?

### 3.2 Instruments

Four instruments were administered via *Survey Tencent*, adapted for cultural relevance:

#### 3.2.1 Sociodemographic information

This part of the questionnaire was designed to collect the following personal variables: gender, age, ethnicity, origin, university location, year of study, and average grade in *Basic/Advanced Spanish*.

#### 3.2.2 Language anxiety

To measure language classroom anxiety in the ELE context, a 12-item questionnaire derived from the Spanish as a Foreign Language Anxiety Scale (Moneypenny and Aldrich, 2022) (e.g., “Tengo miedo de hablar en mi clase de español”) was administered. This shortened version retained the fundamental types of language anxiety associated with learning Spanish as a foreign language. All items were rated on a 7-point Likert scale ranging from 1 (strongly disagree) to 7 (strongly agree). Given that the authors argue that the types of language anxiety should be considered as a whole, no sub-dimensions of this scale were identified. Therefore, according to the suggested interpretation of language anxiety levels, a score of 87% (73 or above on the 84-point scale) indicates a high level of language anxiety; a moderate level is reflected by a score between 64 and 86% (54 to 72); a low level is indicated by a score below 53 (63% or less). The Cronbach’s alpha coefficient supported the reliability of the scale (*α* = 0.95).

#### 3.2.3 Social identity change

To assess the identity change of ELE learners, the Identity Change Scale (Luo, 2019) was used. This questionnaire draws on three classic models related to foreign language learning in a social context: Lambert’s sociopsychological model (1967), Schumann’s acculturation model (1986), and the bilingual model of Gao et al. (2005). The scale used in this study includes four types of identity change, each comprising four items: self-confidence (e.g., “Cuando mi español es mejor que el de otros estudiantes me siento muy bien”), subtractive change (e.g., “Cuando mi nivel de español mejora, empeora mi nivel de chino”), additive change (e.g., “Puedo hablar español y chino en diferentes situaciones contextuales sin ningún problema”), and productive change (e.g., “Después de aprender español me llama más atención el cambio del mundo exterior”). All items were rated on a 7-point Likert scale ranging from 1 (strongly disagree) to 7 (strongly agree), and a higher score in any category indicates a greater degree of identity change experienced by the participant. The Cronbach’s alpha coefficient supported the reliability of the scale (*α* = 0.82).

#### 3.2.4 Expectancy–Value Motivation

For this study, we adapted the Expectancy–Value Motivation Scale for language learning (Spanish version) [originally the English Learning Expectancy–Value Motivation Scale by Dong et al. (2022)], primarily based on the Expectancy–Value Beliefs Inventory (Trautwein et al., 2012). The scale comprises 18 items in total, encompassing five dimensions: expectancy (4 items) (e.g., “Soy bueno en español”), intrinsic value (5 items) (e.g., “Me gustaría tener más clases de español”), achievement value (3 items) (e.g., “La lengua española es importante para mí personalmente”), utility value (3 items) (e.g., “Tengo muchas ganas de aprender mucho en español”), and cost value (3 items) (e.g., “Tendría que invertir mucho tiempo para sacar buenas notas en español”). All items were rated on a 7-point Likert scale ranging from 1 (strongly disagree) to 7 (strongly agree). Higher scores indicate stronger success expectations and subjective values. Reliability analysis indicated that this scale had high internal consistency in the present study (*α* = 0.86).

### 3.3 Procedure and data analysis

The study was conducted in late October 2023, with 421 respondents completing the questionnaire, which consisted of 53 items across 4 dimensions. The questionnaire was administered online through a Chinese survey platform *Survey Tencent*. After reviewing the data, 317 questionnaires were deemed valid, yielding a response rate of 74.6%.

Data analysis was conducted using SPSS v.26. Several statistical analysis methods were applied, including calculations of internal consistency (Cronbach’s alpha), descriptive statistics for the four study variables (mean and standard deviation), correlation analyses among these variables, and multiple and hierarchical linear regression models. It should be noted that the use of the latter regression analysis requires meeting additional assumptions. Accordingly, following Field et al. (2012), we employed the Durbin–Watson test (*p* > 0.05), variance inflation factor (VIF < 4), Q–Q plot, and Cook’s distance (< 1) to verify the independence of errors, check for multicollinearity among predictors, assess the normality of residuals, and identify outliers and influential cases, respectively. Unless otherwise indicated, all assumptions were met.

### 3.4 Ethical considerations

Participation in this study was entirely voluntary and anonymous. No personally identifiable information, such as names, email addresses, or phone numbers, was collected. To further ensure participant privacy, technical identifiers—including IP addresses and device information—were neither recorded nor stored, thereby minimizing the risk of indirect identification. The survey platform was configured to prevent the capture of such metadata. All data were collected and stored in encrypted formats on secure servers, accessible only to the principal investigators. The data are used exclusively for academic research purposes and will not be disclosed to third parties. This study was designed and conducted in accordance with the ethical guidelines of the University of Cordoba and Wenzhou Business College. These measures collectively ensured the confidentiality and integrity of the research process.

## 4 Results

### 4.1 Descriptive and correlational analyses

As shown in Table 1, the sample consisted of 317 Chinese undergraduate students enrolled in Hispanic Philology programs. The gender distribution was unbalanced, with 248 female students (78.2%) and 69 male students (21.8%). Participants ranged in age from 17 to 25 years, with the largest proportion aged 20 (*n* = 69, 21.8%), followed by 22 (*n* = 68, 21.5%) and 21 (*n* = 59, 18.6%). In terms of ethnicity, the majority identified as Han (*n* = 307, 96.8%), while a small minority (*n* = 10, 3.2%) belonged to ethnic minority groups. Regarding the year of study, the most represented group was second-year students (*n* = 119, 37.5%), followed by third-year (*n* = 93, 29.3%), fourth-year (*n* = 67, 21.1%), and first-year students (*n* = 38, 12%). Regarding students’ place of origin, the distribution was relatively balanced across five categories, with the highest proportion from PSGZ regions (*n* = 83, 26.2%). With respect to the location of their university, most were studying in provincial capitals (*n* = 148, 46.7%) or in PSGS pilot zones (*n* = 102, 32.2%). The average Spanish proficiency grade was 75.6 out of 100 (SD = 12.02), with 60 being the minimum passing score. Grade distribution was centered, with 74.1% of students (*n* = 235) scoring between 65 and 85, and 25.9% (*n* = 82) falling outside that range.

**Table 1:** Sociodemographic information of participants.

Variables	Options	*n*	%
1. Age	17	4	1.3
	18	17	5.4
	19	45	14.2
	20	69	21.8
	21	59	18.6
	22	68	21.5
	23	34	10.7
	24	11	3.5
	25	10	3.2
2. Gender	Male	69	21.8
	Female	248	78.2
3. Ethnicity	Han	307	96.8
	Ethnic minorities	10	3.2
4. Current year of study	First year	38	12
	Second year	119	37.5
	Third year	93	29.3
	Fourth year	67	21.1
5.Origin	PSGS	83	26.2
	Provincial capital	72	22.7
	Prefecture-level city	78	24.6
	County-level city	46	14.5
	Town	38	12
6. University location	PSGS	102	32.2
	Provincial capital	148	46.7
	Prefecture-level city	61	19.2
	County-level city	6	1.9
	Town	0	0
7. Spanish achievement	90–100	21	6.6
	85–90	25	7.9
	80–85	72	22.7
	75–80	62	19.6
	70–75	69	21.8
	65–70	32	21.5
	60–65	18	10.1
	50–60	10	3.2
	0–50	8	2.5

n, number of participants; %, percentage of the total sample.

Table 2 presents the means, standard deviations, and correlations for the four variables of the study. Regarding language anxiety in ELE, the majority of participants (*n* = 253, 80.0%) exhibited a low level of anxiety (total scores between 12 and 53), and only 7 students (2.2%) reported a high level of anxiety. Commutatively, nearly 98% of the participants in this study experienced a moderate–low level of language anxiety in the ELE classroom (*M* = 3.37, SD = 1.36). In regards to identity change, self-confidence (*M* = 4.62, SD = 1.08) was highest, followed by additive change (*M* = 4.58, SD = 1.05), productive change (*M* = 4.45, SD = 1.05), and finally subtractive change (*M* = 4.16, SD = 0.95). For Expectancy–Value Motivation, all value-related dimensions were above the mean of expectancy (*M* = 4.63, SD = 1.12). Utility value (*M* = 5.36, SD = 1.16) was the highest among the four value subtypes (achievement value: *M* = 5.03, SD = 1.21; intrinsic value: *M* = 4.98, SD = 1.13; cost value: *M* = 4.72, SD = 1.30).

**Table 2:** Summary of means, standard deviation, and correlations among main variables.

Variables	1	2	3	4	5	6	7	8	9	10	11
1. SA	1	-	–	–	–	–	–	–	–	–	–
2. SFLA	−0.15*	1	–	–	–	–	–	–	–	–	–
3. SC	0.30**	0.21**	1	–	–	–	–	–	–	–	–
4. CS	0.27**	0.15**	0.49**	1	–	–	–	–	–	–	–
5. AC	0.36**	0.00	0.50**	0.59**	1	–	–	–	–	–	–
6. PC	0.24**	0.14*	0.51**	0.56**	0.59**	1	–	–	–	–	–
7. AV	0.44**	−0.14*	0.38**	0.33**	0.40**	0.35**	1	–	–	–	–
8. IV	0.43**	−0.21**	0.41**	0.42**	0.47**	0.48**	0.71**	1	–	–	–
9. VU	0.38**	−0.09	0.48**	0.33**	0.45**	0.42**	0.65**	0.72**	1	–	–
10. VC	0.22**	−0.20**	0.45**	0.37**	0.33**	0.41**	0.37**	0.41**	0.48**	1	–
11. E	0.34**	−0.65**	−0.03	−0.03	0.14*	−0.04	0.33**	0.28**	0.28**	−0.09	1
M	75.6	3.37	4.63	4.16	4.58	4.45	5.03	4.98	5.36	4.72	4.63
ST	12.0	1.35	1.08	0.95	1.05	0.90	1.21	1.13	1.16	1.30	1.12

SA, Spanish achievement; SFLA, Spanish as a foreign language anxiety; CS: Subtractive change; CS, Subtractive change; AC, Additive change; PC, Productive change; AV, Achievement value; IV, Intrinsic value; UV, Utility value; CV, Cost value; E, Expectancy; M, Mean; SD, Standard deviations. ** The correlation is significant at the 0.01 level. * The correlation is significant at the 0.05 level (2-tailed).

As mentioned in the literature on language anxiety in SLA, language anxiety was found to be significantly and negatively related (*r* = −0.15, *p* < 0.01) to the average grade. On the other hand, correlation analyses showed that values and expectancy had a significant (*p* < 0.01) positive correlation with academic performance in Spanish: *r* = 0.44 with achievement value, *r* = 0.43 with intrinsic value, *r* = 0.38 with utility value, *r* = 0.34 with expectancy, and *r* = 0.22 with cost value. Similarly, the various types of identity change were positively intercorrelated, with additive change (*r* = 0.36), self-confidence (*r* = 0.30), subtractive change (*r* = 0.27), and productive change (*r* = 0.24), respectively.

### 4.2 Multiple and hierarchical linear regression models

A multiple and hierarchical linear regression analysis was employed to verify the predictable effects of the three individual differences (language anxiety, identity change, and Expectancy–Value Motivation) of the participants on their academic performance in Spanish. To this end, the means of the three types of Expectancy–Value Motivation, the three types of identity change, and language anxiety were used as predictor variables (requiring inter-correlations among them), while the average grade was used as the dependent variable. In the process of this analysis, three models were generated in Table 3.

**Table 3:** Multiple and hierarchical linear regression models with Spanish achievement as a dependent variable.

	Variables	Unstandardized coefficients		Standardized coefficients			
		*B*	SE	*β*	*t*	*p*	R^2^
Model 1	SC	2.25	0.72	0.20	3.13	**0.00**	0.11
	CS	1.71	0.85	0.14	2.00	0.05	
	AC	0.82	0.91	0.06	0.91	0.37	
Model 2	SC	1.25	0.71	0.11	1.77	**0.01**	
	CS	0.94	0.81	0.07	1.17	0.24	0.24
	AC	−0.41	0.88	−0.03	−0.47	0.64	
	AV	2.6	0.72	0.26	3.60	**0.00**	
	IV	1.96	0.82	0.18	2.40	**0.02**	
	CV	−0.14	0.55	−0.02	−0.26	0.80	
Model 3	SC	1.52	0.71	0.14	2.14	**0.03**	0.25
	CS	1.12	0.81	0.09	1.39	0.17	
	AC	−0.20	0.88	−0.02	−0.23	0.82	
	AV	2.51	0.71	0.25	3.52	**0.00**	
	IV	1.35	0.85	0.13	1.60	0.11	
	CV	0.12	0.55	0.01	0.22	0.83	
	SFLA	−1.1	0.50	−0.13	−2.31	**0.02**	

Independent variables: SC, Self-confidence; CS, Subtractive change; AC, Additive change; AV, Achievement value; IV, Intrinsic value; CV, Cost value; SFLA, Spanish as a foreign language anxiety.

In the first model (M1), self-confidence, subtractive change, and additive change were used to explain Spanish achievement. The model explained 11% of the variance (*R*^2^ = 0.11, *F* = 13.17, *p* < 0.01), indicating a small to moderate overall effect size. Among these variables, self-confidence was the only significant predictor (*β* = 0.20; *p* < 0.01), suggesting that learners who felt more confident in their L2 identity tended to achieve higher grades in Spanish. According to Cohen’s (1988) benchmarks, this represents a moderate effect. Subtractive change and additive change did not reach statistical significance.

In the second model (M2), three EVT dimensions (achievement value, intrinsic value, and cost value) were incorporated as independent variables. This step significantly improved the model fit, increasing the explained variance to 24% (Δ*R*^2^ = 0.13, *F* = 15.92, *p* < 0.01), which constitutes a moderate effect size. Both achievement value (*β* = 0.26, *p* < 0.01) and intrinsic value (*β* = 0.18, *p* < 0.05) significantly predicted Spanish achievement, indicating that students who highly valued success or derived enjoyment from Spanish learning were more likely to perform well. Self-confidence remained marginally significant (*β* = 0.11, *p* = 0.01), suggesting a slight reduction in its effect after introducing motivational factors.

Finally, in the third model (M3), language anxiety was added to examine its additional contribution. In M3, the total variance explained increased slightly to 25% (Δ*R*^2^ = 0.01, *F* = 14.60, *p* < 0.01) and this change represented a small but statistically meaningful improvement. Language anxiety was a significant negative predictor (*β* = −0.13, *p* < 0.05), which showed that students with higher levels of Spanish-related anxiety tended to perform worse academically. Although the effect size was small, it held practical significance, as emotional factors are often negatively associated with language learning outcomes. In the same model, achievement value (*β* = 0.25, *p* < 0.01) and self-confidence (*β* = 0.14, *p* < 0.05) continued to show significant and positive effects on Spanish achievement. These results confirm the meaningful contributions of social identity, EVT, and language anxiety to students’ Spanish achievement.

## 5 Discussion

RQ1 investigates the overall profile of language anxiety, social identity change, and Expectancy–Value Motivation among Chinese university students in the FLE context. The data indicates that participants generally experienced a moderately low level of language anxiety. This finding does not align with previous research in ELE (e.g., Parra, 2016; Rabadán Zurita and Orgambídez Ramos, 2018a,b; Moneypenny and Aldrich, 2022) Such a discrepancy may be attributable to two main factors. First, the participants are students who willingly chose Hispanic Philology as their major. This choice indicates that they clearly have confidence in their linguistic abilities. Second, during the pandemic, relatively strict measures were implemented at Chinese universities between 2020 and 2023. As a result, participants attended more virtual classes than in-person classes. Virtual classrooms can mitigate learners’ communicative pressure and fear of public error, both of which are major sources of language anxiety in face-to-face settings. The combination of intrinsic motivation and reduced real-time interaction may thus explain the relatively low levels of anxiety reported. Despite this, the significant negative correlation between anxiety and academic performance highlights the importance of addressing emotional factors. Even moderate anxiety can hinder performance, which underscore the need for supportive pedagogical strategies.

As for identity change, a positive tendency was observed among participants. The average scores for favorable identity change types were clearly higher than those for the unfavorable, with self-confidence yielding the highest mean score. This observation is consistent with Luo’s (2019) conclusion. Since students chose to study Spanish after the college entrance exam in China, they may be naturally inclined to have a strong interest in the language. Hence, it is not surprising that they maintain or even favor their mother tongue as they delve deeper into learning Spanish as a foreign language.

Regarding EVT, it was found that ELE students at Chinese universities were more motivated by subjective values—particularly driven by utility—and less guided by expectancy in the teaching and learning of ELE, which is consistent with previous research in China (Wan, 2019; Dong et al., 2022). Once again, this result highlights that Chinese (and other Asian) students of Spanish, like those who study English as a foreign language, consider that learning a foreign language is highly useful for achieving their future goals, such as finding a suitable job or being admitted into a prestigious university. However, a notable finding in this study is that students also reported high levels of achievement value and intrinsic value, which suggests that they seek not only instrumental goals but also personal growth. Unlike English, which is widely studied and institutionally promoted, Spanish represents a more self-chosen and identity-invested path. For these students, performing well in Spanish may serve to reinforce self-confidence and competence, especially given the perceived difficulty of mastering an additional foreign language. The prominence of achievement value thus underscores the importance of learners’ desire to excel and prove their capabilities in a challenging academic context.

RQ2 examines how language anxiety, identity change, and Expectancy–Value Motivation predicted the academic performance in Spanish of ELE students at Chinese universities. As shown in Table 1, Spanish grades were positively correlated with identity change and Expectancy–Value Motivation, whereas academic performance in Spanish inversely correlated to language anxiety in the ELE context. These results are also consistent with the most recent literature (Rabadán Zurita and Orgambídez Ramos, 2018b; Dong et al., 2022; Moneypenny and Aldrich, 2022).

The multiple and hierarchical regression models (see Table 2) indicated that Chinese students’ average Spanish grade is significantly predicted by achievement value, self-confidence, and language anxiety. Achievement value was the only type of motivation that positively predicted participants’ performance in Spanish, which contradicts previous findings that identified utility value and intrinsic value as the most influential factors (Loh, 2019; Dong et al., 2022). It is likely that these ELE students aim to learn Spanish to demonstrate their linguistic competence, since learning a new language from scratch is neither easy nor necessarily enjoyable, especially given their long-standing experience with English as a required subject since primary school.

As for the other two factors, the findings are consistent with existing conclusions, despite the participants’ relatively mild level of language anxiety. This suggests that it is crucial to implement appropriate pedagogical strategies to reduce language anxiety levels among ELE learners (Rabadán Zurita and Orgambídez Ramos, 2018b).

## 6 Conclusion

This study provided an overall profile of Expectancy–Value Motivation, language anxiety and social identity among Chinese undergraduates majoring in Hispanic Philology. It explored the predictive effects of these factors on their academic performance in Spanish. The results indicated that the Chinese ELE participants had a relatively higher level of value-based motivation compared to their level of expectancy. Although utility value had the highest mean score, achievement value was the only motivational factor that positively predicted performance in Spanish. Additionally, the Chinese undergraduates surveyed showed an overall positive tendency toward identity change, particularly when it comes to self-confidence, which was the predominant type and a positive predictor. Finally, Chinese students exhibited a moderately low level of language anxiety in the ELE classroom. Likewise, this was the only variable that negatively predicted their Spanish achievement.

In spite of the previous findings, this study had some limitations. Firstly, theoretically speaking, this study examined only classroom language anxiety because of the specific anxiety scale employed. However, it should be noted that this negative emotion can also be generated outside of the classroom (Jiang and Dewaele, 2020), so future research could study this type of anxiety in further detail. Secondly, this study used the average grade in one key subject from the Hispanic Philology program as the measure of academic performance in Spanish, but that score does not fully reflect the students’s overall Spanish proficiency or specific language skills. For this reason, future studies may assess specific language competencies (e.g., oral or written proficiency) by employing task-based learning approaches (Li et al., 2016). In third place, it is worth noting that the findings of the present study are based exclusively on cross-sectional data; therefore, it cannot be asserted that they reflect causal relationships between the variables measured. Lastly, although several sociodemographic factors were collected (e.g., gender, university location), their relationships with language outcomes and individual differences were not examined. Future studies may explore these variables more systematically and consider longitudinal or experimental designs to address causality.

Despite these limitations, this study deepens the literature on how these individual differences—both positive and negative—interrelate in the process of learning Spanish as a foreign language in Chinese universities. It is expected that further empirical research on this topic will be conducted, incorporating other emotions, to fill this gap in the context of Spanish as a foreign language.

## Data Availability

The original contributions presented in the study are included in the article/supplementary material, further inquiries can be directed to the corresponding author.

## References

[ref1] Aida Y. (1994). Examination of Horwitz, Horwitz, and Cope’s construct of foreign language anxiety: the case of students of Japanese. Mod. Lang. J. 78, 155–168. doi: 10.1111/j.1540-4781.1994.tb02026.x

[ref2] Al-Hoorie A. H. (2018). The L2 motivational self system: a meta-analysis. Stud. Second Lang. Learn. Teach. 8, 721–754. doi: 10.1111/ijal.12416

[ref3] Almarza A. Z. M. López I. Díaz J. A. M. (2012). Role influencia de la distancia social en el aprendizaje de lenguas extranjeras (LE). Encuent. Educ. 19, 180–194.

[ref4] Atkinson J. W. (1957). Motivational determinants of risk-taking behavior. Psychol. Rev. 64, 359–372. doi: 10.1037/h0043445, PMID: 13505972

[ref5] Busse V. (2017). Plurilingualism in Europe: exploring attitudes toward English and other European languages among adolescents in Bulgaria, Germany, the Netherlands, and Spain. Mod. Lang. J. 101, 566–582. doi: 10.1111/modl.12415

[ref9005] Instituto Cervantes. (2024). El español en el mundo. Anuario del Instituto Cervantes 2024. Madrid: Instituto Cervantes.

[ref6] Clouet R. (2020). Influencia de la cultura y de la identidad en el aprendizaje de una lengua extranjera: aplicación del modelo de las seis dimensiones de Hofstede (2010) para analizar el proceso de aprendizaje de ELE por parte de estudiantes surcoreanos. Onomázein 47, 46–79. doi: 10.7764/onomazein.47.03

[ref9001] Cohen J. (1988). Statistical Power Analysis for the Behavioral Sciences (2nd ed.). Hillsdale, NJ: Lawrence Erlbaum Associates, Publishers.

[ref7] Darvin R. Norton B. (2015). Identity and a model of investment in applied linguistics. Annu. Rev. Appl. Linguist. 35, 36–56. doi: 10.1017/S0267190514000191

[ref8] Dewaele J.-M. Al-Saraj T. M. (2015). Foreign language classroom anxiety of Arab learners of English: the effect of personality, linguistic and sociobiographical variables. Stud. Second Lang. Learn. Teach. 5, 205–228. doi: 10.14746/ssllt.2015.5.2.2

[ref9] Dewaele J.-M. Li C. (2022). Foreign language enjoyment and anxiety: associations with general and domain-specific English achievement. Chin. J. Appl. Linguist. 45, 32–48. doi: 10.1515/CJAL-2022-0104

[ref10] Doğan Y. Tuncer M. (2016). Examination of foreign language classroom anxiety and achievement in foreign language in Turkish university students in terms of various variables. J. Educ. Train. Stud. 4, 22–34. doi: 10.11114/jets.v4i5.1337, PMID: 39100657

[ref11] Dong L. Liu M. Yang F. (2022). The relationship between foreign language classroom anxiety, enjoyment, and expectancy-value motivation and their predictive effects on Chinese high school students’ self-rated foreign language proficiency. Front. Psychol. 13:860603. doi: 10.3389/fpsyg.2022.860603, PMID: 35712204 PMC9196878

[ref9002] Dörnyei Z. (2008). The Psychology Of The Language Learner: Individual Differences In Second Language Acquisition. Mahwah, NJ: Lawrence Erlbaum.

[ref12] Dörnyei Z. (2009). “The L2 motivational self system” in Motivation, language identity and the L2 self. eds. Dörnyei Z. Ushioda E. (Bristol: Multilingual Matters), 9–42.

[ref13] Eccles J. S. Wigfield A. (2020). From expectancy-value theory to situated expectancy-value theory: a developmental, social cognitive, and sociocultural perspective on motivation. Contemp. Educ. Psychol. 61:101859. doi: 10.1016/j.cedpsych.2020.101859

[ref14] Fernández Sánchez E. (2018). “La ASL desde una aproximación psicolingüística” in Teoría y metodología para la enseñanza de ELE. I, Fundamentos, enfoques y tendencias. eds. Martínez-Atienza de Dios M. Zamorano Aguilar A. (Madrid: enClave-ELE), 13–37.

[ref9003] Field A. Miles J. Field Z. (2012). Discovering Statistics Using R. London: Sage Publications Ltd.

[ref15] Gao Y. Zhao L. Cheng Y. Zhou Y. (2005). Self-identity changes and English learning among Chinese undergraduates. World Engl. 24, 39–51. doi: 10.1111/j.0883-2919.2005.00386.x, PMID: 40674112

[ref16] Gardner R. C. (1985). Social psychology and second language learning: the role of attitudes and motivation. London: Edward Arnold.

[ref17] Gardner R. C. (2007). Motivation and second language acquisition. Porta Linguarum 8, 9–20. doi: 10.30827/Digibug.31616, PMID: 40520326

[ref18] Gregersen T. MacIntyre P. D. Meza M. D. (2014). The motion of emotion: idiodynamic case studies of learners’ foreign language anxiety. Mod. Lang. J. 98, 574–588. doi: 10.1111/modl.12084, PMID: 40674112

[ref9004] Horwitz E. K. (2017). “On the Misreading of Horwitz, Horwitz and Cope (1986) and the Need to Balance Anxiety Research and the Experiences of Anxious Language Learners,” in New Insights into Language Anxiety: Theory, Research and Educational Implications, eds. Gkonou C. Daubney M. Dewaele J.-M. (Bristol, Blue Ridge Summit: Multilingual Matters), 31–48.

[ref19] Horwitz E. K. Horwitz M. B. Cope J. (1986). Foreign language classroom anxiety. Mod. Lang. J. 70, 125–132. doi: 10.2307/327317

[ref20] Jiang Y. Dewaele J.-M. (2020). The predictive power of sociobiographical and language variables on foreign language anxiety of Chinese university students. System 89:102207. doi: 10.1016/j.system.2020.102207

[ref21] Lambert W. E. (1967). A social psychology of bilingualism. J. Soc. 23, 91–109. doi: 10.1111/j.1540-4560.1967.tb00578.x, PMID: 40674112

[ref22] Li S. Ellis R. Zhu Y. (2016). “Individual differences in second language acquisition: Theory, research, and practice,” in The Routledge Handbook of Second Language Acquisition and Individual Differences, ed. S. Li (New York, NY: Routledge), 3–34.

[ref23] Li S. Hiver P. Papi M. (2022). “Individual differences in second language acquisition: theory, research, and practice” in The Routledge handbook of second language acquisition and individual differences (New York, NY: Routledge), 3–34.

[ref24] Loh E. K. (2019). What we know about expectancy-value theory, and how it helps to design a sustained motivating learning environment. System 86:102119. doi: 10.1016/j.system.2019.102119

[ref25] Lou N. M. Noels K. A. (2020). Mindsets matter for linguistic minority students: growth mindsets foster greater perceived proficiency, especially for newcomers. Mod. Lang. J. 104, 739–756. doi: 10.1111/modl.12669

[ref27] Luo C. (2019). Un estudio correlacional sobre la motivación e identidad de los licenciados chinos de español y la propuesta didáctica de mejora para el ELE en China. Madrid: Universidad Complutense de Madrid.

[ref26] Lu Q. (2023). El modelo de la aculturación y adquisición del español como L2: Un estudio correlacional de estudiantes sinohablantes del grado en Filología Hispánica. Córdoba: Universidad de Córdoba.

[ref28] MacIntyre P. D. (2017). “An overview of language anxiety research and trends in its development” in New insights into language anxiety: theory, research and educational implications. eds. Gkonou C. Daubney M. Dewaele J.-M. (Bristol: Multilingual Matters), 11–30.

[ref29] MacIntyre P. D. Gardner R. C. (1994). The subtle effects of language anxiety on cognitive processing in the second language. Lang. Learn. 44, 283–305. doi: 10.1111/j.1467-1770.1994.tb01103.x

[ref30] Minera L. E. (2010). La motivación y las actitudes de aprendizaje del E/LE en los estudiantes no hispanistas de la Universidad LMU de Múnich. Rev. Nebrija Ling. Apl. Enseñ. Lenguas 8, 42–80. doi: 10.26378/rnlael48141

[ref31] Moneypenny D. B. Aldrich R. S. (2022). Foreign language anxiety in online college Spanish: prevalence and effects on oral proficiency. Lang. Teach. Res. 29, 2245–2262. doi: 10.1177/13621688221112378, PMID: 40672049

[ref32] Morales K. I. Morales Cabezas J. (2021). Motivación y competitividad: Un estudio en el aula de japonés como lengua extranjera. Porta Linguarum 35, 125–132. doi: 10.30827/portalin.vi35.15724

[ref33] Nagle C. (2021). Using expectancy value theory to understand motivation, persistence, and achievement in university-level foreign language learning. Foreign Lang. Ann. 54, 1238–1256. doi: 10.1111/flan.12569

[ref34] Norton B. (2013). Identity and language learning: extending the conversation. Bristol: Multilingual Matters.

[ref35] Norton B. Gao Y. (2008). Identity, investment, and Chinese learners of English. J. Asian Pac. Commun. 18, 109–120. doi: 10.1075/japc.18.1.07nor

[ref36] Norton Peirce B. (1995). Social identity, investment, and language learning. TESOL Q. 29, 9–31. doi: 10.2307/3587803

[ref37] Oga-Baldwin W. L. Q. Fryer L. K. Larson-Hall J. (2019). The critical role of the individual in language education: new directions from the learning sciences. System 86:102118. doi: 10.1016/j.system.2019.102118

[ref38] Ortega L. (2009). Understanding second language acquisition. New York: Routledge.

[ref39] Oxford R. (2015). Emotion as the amplifier and the primary motive: some theories of emotion with relevance to language learning. Stud. Second Lang. Learn. Teach. 5, 371–393. doi: 10.14746/ssllt.2015.5.3.2

[ref9006] Park G. P. (2014). Factor analysis of the foreign language classroom anxiety scale in Korean learners of English as a foreign language. Psychol. Rep. 115, 261–275. doi: 10.2466/28.11.PR0.115c10z2 25153961

[ref40] Parra F. J. F. (2016). La ansiedad ante las destrezas orales en la clase de español lengua extranjera: una propuesta blended-learning con sinohablantes. Rev. Ling. Leng. Apl. 11, 19–33. doi: 10.4995/rlyla.2016.4445

[ref41] Rabadán Zurita M. Orgambídez Ramos A. (2018a). Ansiedad idiomática en español como lengua extranjera y rendimiento académico en la enseñanza superior. Rev. Estud. Investig. Psicol. Educ. 5, 29–35. doi: 10.17979/reipe.2018.5.1.2905

[ref42] Rabadán Zurita M. Orgambídez Ramos A. (2018b). Ansiedad idiomática y motivación hacia el español como lengua extranjera en estudiantes universitarios portugueses: un estudio exploratorio. Estud. Sobre Educ. 35, 517–533. doi: 10.15581/004.34.517-533

[ref44] Rubio F. (2004). La ansiedad en el aprendizaje de idiomas. Huelva: Servicio de Publicaciones de la Universidad de Huelva.

[ref45] Schumann J. H. (1986). Research on the acculturation model for second language acquisition. J. Multiling. Multicult. Dev. 7, 379–392. doi: 10.1080/01434632.1986.9994254

[ref46] Shao K. Pekrun R. Nicholson L. J. (2019). Emotions in classroom language learning: what can we learn from achievement emotion research? System 86:102121. doi: 10.1016/j.system.2019.102121, PMID: 40683280

[ref9007] Teimouri Y. Goetze J. Plonsky L. (2019). Second language anxiety and achievement: A meta-analysis. Stud. Second Lang. Acquis. 41, 363–387. doi: 10.1017/S0272263118000311

[ref9009] Toyama M. Yamazaki Y. (2018). Exploring the components of the foreign language classroom anxiety scale in the context of Japanese undergraduates. Asian J. Second. Foreign Lang. Educ. 3, 4. doi: 10.1186/s40862-018-0045-3

[ref9008] Trautwein U. Marsh H. W. Nagengast B. Lüdtke O. Nagy G. Jonkmann K. (2012). Probing for the multiplicative term in modern expectancy–value theory: A latent interaction modeling study. J. Educ. Psychol. 104, 763–777. doi: 10.1037/a0027470

[ref47] Wada H. (2021). La ansiedad de lenguas extranjeras de los estudiantes japoneses de ELE en un país hispanohablante: Una comparación con los aprendientes en Japón. HISPÁNICA 2021, 55–79. doi: 10.4994/hispanica.2021.55

[ref48] Wan Z. H. (2019). Exploring the effects of intrinsic motive, utilitarian motive, and self-efficacy on students’ science learning in the classroom using the expectancy-value theory. Res. Sci. Educ. 51, 647–659. doi: 10.1007/s11165-018-9811-y, PMID: 40682715

[ref49] Wigfield A. Eccles J. S. (2000). Expectancy–value theory of achievement motivation. Contemp. Educ. Psychol. 25, 68–81. doi: 10.1006/ceps.1999.1015, PMID: 10620382

[ref50] Woodrow L. (2006). Anxiety and speaking English as a second language. RELC J. 37, 308–328. doi: 10.1177/0033688206071315

[ref51] Young D. J. (1991). Creating a low-anxiety classroom environment: what does language anxiety research suggest? Mod. Lang. J. 75, 426–439. doi: 10.2307/329492, PMID: 39964225

[ref52] Zhang X. (2019). Foreign language anxiety and foreign language performance: a meta-analysis. Mod. Lang. J. 103, 763–781. doi: 10.1111/modl.12590

